# Safety and Efficacy of Surgical Techniques in Treating Lipedema: Systematic Review

**DOI:** 10.1093/asjof/ojag039

**Published:** 2026-02-24

**Authors:** José Alejandro Acuña Vengoechea, Ricardo Coronel Gagliardi, María Isabel Manzano Martín, Camilo Zuleta Valencia, Benito Madiedo Triana, Sonia Blanco Limia, Cristian Rojas Figueroa, Curro Millán, Patrícia Froes Meyer

## Abstract

Lipedema is a chronic disorder characterized by abnormal subcutaneous fat accumulation, mainly in women's lower limbs. The aim of the study was to analyze the safety and efficacy of liposuction in treating lipedema. A systematic review was conducted in PubMed, Scopus, and MEDLINE until June 2024 using the terms “lipedema,” “liposuction,” “results,” and “complications.” Twenty-five studies were included (*n* = 2373 patients). Liposuction, mainly using the tumescent infiltration, reduced pain, BMI, and functional limitations, with improvements in mobility and quality of life. Mean aspirated fat volume was 3077 mL per session and 6111 mL per treatment course. Complications were uncommon (hematomas, edema, anemia, DVT, and rare methemoglobinemia). Moreover, improvements were maintained during the follow-up, suggesting that the beneficial results were sustained over time. Although 15 studies reported using tumescent, only 2 fully described the anesthetic fluid composition. Liposuction is a safe and effective treatment for lipedema, but variability in techniques and postoperative care highlights the need for standardized protocols and further research.

**Level of Evidence**: 4 (Therapeutic)

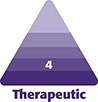

Lipedema is a chronic condition characterized by the abnormal accumulation of subcutaneous fat, mainly affecting the lower limbs of women. Although this condition is often confused with lymphedema, obesity, and cellulitis, lipedema has specific clinical characteristics that allow differential diagnosis.^1^

The pathophysiology of lipedema involves genetic, hormonal, metabolic, and microcirculatory factors. The genetic predisposition is notable because the condition often occurs in several women within the same family, affecting ∼60.0% of patients who have an affected first-degree relative. Hormonal changes (eg, puberty, pregnancy, and menopause) are also related to lipedema, indicating that female sex hormones (eg, estrogen) influence the pathophysiology.^2,3^

The abnormal accumulation of subcutaneous fat caused by lipedema does not respond to diet and exercise. Affected fat cells may show metabolic changes (ie, insulin resistance and chronic local inflammation) that result in pain and altered sensitivity. Changes in microcirculation, such as fragile capillaries and increased permeability of blood vessels, are common and lead to interstitial edema and hematoma. Also, chronic low-grade inflammation worsens these symptoms.^4,5^

The diagnosis of lipedema remains primarily clinical and is based on history and physical examination, with particular importance in differentiating lipedema from conditions, such as obesity and lymphedema.^6^ Key clinical features include a characteristic, symmetric distribution of subcutaneous adipose tissue affecting the limbs (typically sparing the hands and feet), easy bruising, nodularity of the subcutaneous tissue, and increased tenderness/pain on palpation or with pressure. Although staging systems (Stages 1-4) are primarily morphological, describing the progression from increased subcutaneous tissue and small nodularity (Stage 1) to lobular deformity and secondary lymphatic involvement (Stage 4), pain and sensitivity are core clinical features that contribute to the diagnosis, patient burden, and treatment decision making. Imaging (eg, ultrasound or MRI) may assist in differential diagnosis but is not required for clinical diagnosis in most cases.^3,7^

Lipedema is classified into different stages according to the severity of the clinical manifestations^3,4,7^: Stage 1: Smooth skin with increased subcutaneous tissue, but several small nodules can be palpated. Stage 2: Skin with irregular and harder appearance because of increased nodular structure (large nodules) of subcutaneous fat (liposclerosis). Stage 3: Lobular deformation of the skin surface because of increased fat. The nodules vary in size and can be distinguished from surrounding tissue on palpation. Stage 4: Large deformity of skin and fat associated with damage to the lymphatic system, causing edema in the feet (known as lipo-lymphedema). The progression to lipedema with secondary lymphedema can occur at any stage.

Liposuction can be described according to 2 different technical axes: (A) the suction/energy modality (eg, suction-assisted liposuction [SAL], power/energy-assisted methods such as energy-assisted liposuction [EAL]/ultrasound-assisted liposuction [UAL]/laser-assisted liposuction [LAL], or water jet–assisted liposuction [WAL]) and (B) the method of fluid infiltration used prior to aspiration (commonly tumescent infiltration). It is important to emphasize that tumescent infiltration (delivery of a local anesthetic/vasoconstrictive solution into the tissues) is a fluid infiltration strategy that can be used with most suction modalities and therefore should not be considered a separate suction technique. The main suction/energy modalities are: SAL (manual aspiration), EAL (power-assisted cannulas), UAL (ultrasound energy to emulsify fat), LAL (laser energy), and WAL (pulsatile water jet). Each suction modality has specific physical mechanisms and potential advantages or drawbacks, similarly, infiltration strategies vary in composition and volume and may influence outcomes, but the infiltration method is conceptually distinct from the suction modality itself.^8-10^

Several factors should be considered when choosing a liposuction technique to treat lipedema (such as the extent of the condition, patient characteristics, and surgeon preferences). Each technique offers specific advantages, and a proper selection may improve results and recovery. Emerging techniques continue to expand available options, enabling more personalized and effective treatments for managing lipedema.^11^ Despite the increasing use of liposuction to treat symptomatic lipedema, there is limited systematic evidence directly comparing outcomes between different suction/energy modalities and between different infiltration strategies (tumescent vs nontumescent approaches). This review therefore aims to synthesize the available evidence on the safety and efficacy of surgical approaches for lipedema, with particular attention to specifying which suction modality and infiltration method were used in each included report.

## METHODS

This review was conducted according to Preferred Reporting Items for Systematic Reviews and Meta-Analyses (PRISMA) guidelines (Appendix).^12^ This review was not registered in PROSPERO.

### Literature Search

For this systematic review, the electronic databases PubMed, Scopus, and MEDLINE were searched until June 30, 2024, to identify studies that reported surgical techniques for lipedema. Research strategy used was (“lipedema” [Mesh] OR “lipoedema” [tiab] OR “lipedema” [tiab]) AND (“Liposuction” [Mesh] OR “liposuction” [tiab] OR “lipectomy” [tiab] OR “liposuction surgery” [tiab]) AND (“Treatment Outcome” [Mesh] OR “outcome” [tiab] OR “results” [tiab] OR “complication” [tiab]).

The search captured review articles, case reports, original research articles, and any other articles relevant to lipedema. Article reference lists were also examined for applicable studies. Review articles were also screened to ensure no primary study was missed. In addition, review articles were screened to identify any primary studies not captured by the database search. We additionally screened reference lists of included studies and relevant reviews.

### Inclusion Criteria and Outcome Measures

Studies were included if they reported adult patients (≥18 years) undergoing surgical treatment for lipedema reporting safety or efficacy outcomes. Exclusion criteria: reviews, editorials, commentaries, conference abstracts, technical reports, studies with pediatric populations (<18 years), studies of nonsurgical treatments, or studies lacking relevant outcome data. Studies assessed with the Newcastle–Ottawa Scale (NOS) <5 were excluded a priori.^13^ No language restrictions were applied.

The primary outcomes assessed were (1) improvement in pain symptoms, (2) reduction of limb volume or circumference, (3) functional improvement (mobility, gait, and activities of daily living), (4) quality-of-life scores, and (5) postoperative complications. Secondary outcomes included aspirated volume per session, number of procedures, and long-term recurrence or need for additional conservative therapy.

### Study Selection and Data Extraction

Duplicate records were removed prior to screening. Titles and abstracts were then evaluated to assess eligibility, followed by a full-text review of all potentially relevant articles to determine whether they met the predefined inclusion criteria. Two independent reviewers (P.F.M. and M.I.M.M.) screened all studies and extracted data, and disagreements were resolved through consultation with a third reviewer (J.A.A.V.). In addition, reference lists of all included articles were manually reviewed to identify additional eligible studies.

All included studies were observational in design (prospective or retrospective cohorts, cross-sectional analyses, or case series); therefore, the NOS was applicable to all of them, and no additional risk-of-bias tools were required. The methodological quality of each study was independently assessed by 2 reviewers using the NOS, which evaluates 3 domains: selection of study groups, comparability of groups, and ascertainment of outcomes. Studies scoring <5 out of 9 were considered high risk of bias and excluded a priori. This threshold was selected to avoid incorporating studies with major methodological limitations while still permitting moderate-quality studies (Scores 5-6) to contribute evidence. Discrepancies in scoring were resolved by consensus.

Data extraction followed a structured form and included study design, sample size, patient characteristics, staging, anatomical region treated, liposuction modality, tumescence details, operative parameters, complications, and postoperative outcomes. When possible, the number of patients contributing to each reported outcome was extracted. However, several studies did not provide outcome-specific denominators; in these cases, results are presented as the number of studies reporting the outcome rather than a pooled patient denominator. Missing data were recorded as “not reported” and indicated in the Supplemental Tables. All data entries were double-checked for accuracy. For clarity, a “session” was defined as a single operative event (often addressing one or more anatomic regions in the same anesthetic), whereas a “treatment course” may comprise multiple sessions per patient.

### Statistical Analysis

Extracted data were synthesized using descriptive statistics. Continuous variables (eg, age, BMI, aspirated volume) were summarized as means or ranges as reported by the original studies. Categorical variables (eg, complications, staging, and liposuction modality) were summarized using absolute numbers and percentages. Given the substantial heterogeneity in study designs, populations, outcome definitions, and reporting formats, no meta-analysis or pooled effect estimation was performed. Instead, findings were narratively synthesized according to liposuction technique, tumescent protocol, and clinical outcomes.

## RESULTS

**Figure 1.**
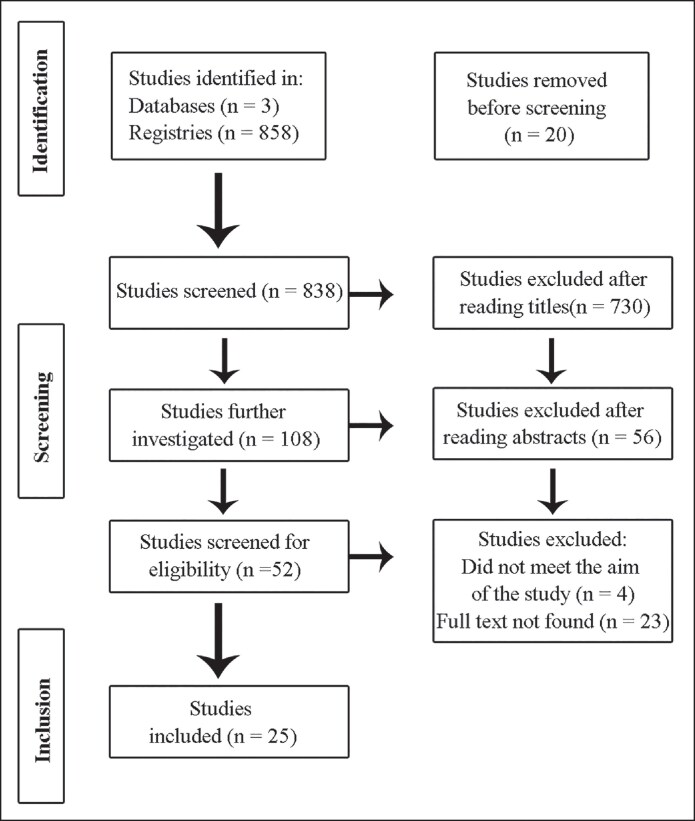
PRISMA flowchart of studies selection.

The search found 858 studies (412 from PubMed, 358 from Scopus, and 88 from Medline). After duplicate removal (*n* = 20), 838 studies were selected. Next, title screening excluded 730 studies. Of the 108 studies that had their abstracts analyzed, 52 were read in full, and 25 were included.^14-38^ Of the 25 included studies, 14 were retrospective cohorts, 6 were prospective cohorts, 3 were cross-sectional analyses, and 2 were case series. No randomized or comparative trials were identified. Figure 1 shows the PRISMA flowchart for study selection. Duplicates were excluded, and full-text studies were analyzed considering the inclusion criteria.

A total of 25 studies comprising 2373 patients were included. Tumescent anesthesia was used in 15 studies (60%), and only 10 of them described tumescence as the primary operative technique (*n* = 704 patients). The remaining studies used WAL, EAL, UAL, or LAL with tumescent infiltration as an adjunct rather than the main technique. WAL was reported in 4 studies (*n* = 290), UAL in 1 study (*n* = 112), and EAL in 2 studies (*n* = 112). LAL was used in 1 study (*n* = 3) and conventional SAL in 2 isolated cases (*n* = 2). Five studies (*n* = 1058) involved mixed or not fully specified techniques. Detailed study-level information is provided in Supplemental Table 1, and quality assessment is summarized in Supplemental Table 2 and Supplemental Figure 1.

### Risk of Bias

We assessed methodological quality using the NOS. A study-level summary is provided in Supplemental Table 2. For transparency, 15 studies (NOS ≥7) were judged at low risk of bias and 10 studies (NOS 5-6) at moderate risk. No study scored below 5; therefore, none was excluded a priori. Most limitations were related to incomplete outcome reporting, lack of controls, and heterogeneity of follow-up. A visual representation is provided in Supplemental Figure 1.

### Patient Profile and Basic Characteristics

Most studies were retrospective (*n* = 8); however, 1 study included a retrospective and a prospective phase, and 4 studies were prospective cohorts.^14-18,20,21,23,24,27,29,30,36^ Among the observational designs, 3 studies were cross-sectional and 1 was a comparative study.^19,22,34,35^ Also, 3 case studies, including one with histological and immunohistochemical analysis, and 5 case series were analyzed.^25,26,28,31-33,37,38^

A total of 25 studies comprising 2373 patients (100%) were included. Sample sizes ranged from 20 to 645 participants per study. Female patients represented the vast majority (*n* = 2345/2373; 98.9%), with only 27 male patients (1.1%) reported. Preoperative BMI was reported in 14 studies (*n* = 1620/2373; 68.3%). The average BMI ranged from 26.4 to 39.5 kg/m^2^. Postoperative BMI was reported in 3 studies (*n* = 215/2373; 9.1%), with a mean value of 34.7 kg/m^2^.

All patients presented lipedema in the lower limbs (*n* = 21/25; 84.0%), in internal and external areas (thigh, calf, and ankle). Rapprich et al described 20 patients with lipedema affecting the entire leg, whereas 3 were affected only in the thigh and 2 only in the lower leg.^27^ Also, according to Rapprich et al, the locations most affected by lipedema in 85 patients were the entire leg (62.4%), followed by the thigh (30.6%), and the calf (6.0%), respectively.^34^ Only 4 studies (*n* = 325/2373; 13.7%) included patients with upper limb involvement.

### Interventions and Comparators

Regarding liposuction modality, WAL was used as the primary technique in 4 studies (*n* = 290).^24-26,35^ EAL was used in 2 studies (*n* = 112) and UAL in 1 study (*n* = 112).^21,26,34^ LAL was used in a study (*n* = 3).^32^ Conventional SAL appeared in 2 isolated case reports (*n* = 2).^27,28^ Five studies reported mixed or not fully specified techniques (*n* = 1058).^18,19,23,29,31^ In total, 14 studies clearly described their operative technique, whereas 2 did not provide sufficient detail.

Across 15 studies (*n* = 1425 patients; 60.1%), the mean number of treatment sessions per patient was 3.13 (range, 1-12). Wollina and Heinig reported 111 patients undergoing 334 sessions (mean ≈3.01 sessions per patient).^20^ The mean aspirated fat volume was 3077 mL per session and 6111 mL per treatment course, reported in 12 studies (*n* = 980/2373; 41.3%).

Only 3 studies explicitly reported preoperative laboratory testing or specific prophylactic measures, 3 studies reported tests or prophylactic measures for patient monitoring during the preoperative period.^18,20,38^ Wright et al analyzed the presurgical mapping of the main superficial veins and lymphatic structures at surgical sites (arms or legs).^18^ Wollina and Heinig analyzed glucose-6-phosphate and methemoglobin deficiency before the treatment course.^20^ Wollina et al provided a single intravenous injection of ascorbic acid for all patients before the treatment course to reduce the risk of methemoglobin formation.^38^

### Preoperative Measures

Pharmacologic prophylaxis with low molecular weight heparin was explicitly reported in 2 studies (*n* = 120 patients; 5.1% of the total review population). In these, enoxaparin 40 mg once daily was administered for 5 to 10 days postoperatively. Other included reports variably mentioned prophylaxis but did not provide denominators. Compression garments were reported in 10 studies, although duration of use ranged from 2 weeks to 6 months. However, Peled et al recommended using compression garments for 6 months postoperatively.^31^ Manual lymphatic drainage was described as an adjunct in several studies, typically beginning 2 to 3 weeks postoperatively.^21,24,27,34^

Other studies also used antibiotic therapy for 3 days: ciprofloxacin 2 × 250 mg or cefuroxime 2 × 250 mg, ciprofloxacin 2 × 500 mg, amoxicillin or erythromycin 3 × 500 mg, or a single injection only for perioperative prophylaxis.^21,24,27,33-35^ This was reported in 4 studies involving *n* = 203 patients, additionally, Navadeh also mentioned the use of antibiotic prophylaxis, but the number of patients was not explicitly reported.^33^ In Witte et al, all 63 patients received perioperative prophylaxis according to the protocol.^24^

### Patient-Reported Outcomes and Complications

Volume or limb circumference reduction was assessed in 10 studies (*n* = 1284 patients).^15,17,18,20,23-25,29,30,32^ Across the included studies, WAL was used in 5 studies (*n* = 532 patients), SAL in 4 studies (*n* = 474 patients), and UAL in 1 study (*n* = 278 patients).^17,18,20,22,23,27-30,33^ All studies reported postoperative reductions in limb volume or circumference, which were generally sustained in medium- and long-term follow-up.

Quality of life was reported in 13 studies (*n* = 1845 patients).^14,15,17-19,21-23,27-29,31,34^ WAL was used in 6 studies (*n* = 658 patients), SAL in 5 studies (*n* = 511 patients), UAL in 1 study (*n* = 128 patients), and LAL in 1 study (*n* = 38 patients).^17,19,21-23,27-34^ Across all modalities, improvements were documented in physical functioning, daily activities, and psychosocial well-being. Thus, the number of studies per technique differs from the overall distribution because not all studies using a given technique reported QoL.

Occupational disabilities also impaired the lives of patients with lipedema. After liposuction, 86.0% of patients reported improvement or complete recovery from disabilities.^14,20^ About 58.0% of patients reported needing less or no disease attestation because of lipedema-related symptoms after liposuction. With a median global satisfaction of 90 points (0-100), 99.0% of all patients would recommend the multistage surgical therapy.^29^

Liposuction was generally considered a safe treatment course for treating lipedema. In this review, complications were reported in 251 of 2373 patients (10.6%) across 12 studies. The most frequently described adverse events were: edema (*n* = 145/2373; 6.1%), postoperative hematomas (*n* = 32/2373; 1.3%), postoperative anemia (*n* = 18/2373; 0.8%), deep vein thrombosis (*n* = 6/2373; 0.3%), skin changes (*n* = 22/2373; 0.9%), and infections (*n* = 10/2373; 0.4%). Two studies, specifically addressed the risk of methemoglobinemia, an uncommon but potentially serious complication associated with local anesthesia.^20,38^ No cases were reported in their cohorts (*n* = 111 and *n* = 30 patients, respectively), possibly reflecting the prophylactic strategies adopted, such as dose adjustment of lidocaine and careful monitoring. Wollina and Heinig reported outcomes in 111 patients (334 treatment courses) with low rates of adverse events, whereas Witte et al observed favorable safety in 63 patients treated with a standardized WAL protocol.^20,24^

### Pain Outcomes

Pain or pressure sensitivity was explicitly evaluated in 15 of the 25 included studies (*n* = 2127 patients).^20-38^ Measures of pain varied between studies (visual analog scales, symptom questionnaires), and reporting of follow-up time points was heterogeneous. Across studies, clinically relevant pain reduction was observed early (within weeks to months after surgery) and was sustained in medium-term follow-up (≥12 months) in several cohorts. Importantly, long-term observational series reported maintained benefit: Dadras et al described sustained symptom relief 8 years postoperatively in 25 patients, Schmeller et al demonstrated significant pain reduction up to 12 years in 25 patients, and Schmeller and Meier-Vollrath reported consistent improvements in 164 patients with follow-up up to 8 years.^21,22,28^ Additionally, Baumgartner et al reported persistent improvement in spontaneous pain, pressure sensitivity, and functional limitations after 4 and 8 years in 28 patients.^20^ Because primary reports used heterogeneous instruments and did not converge on a single follow-up time point, we present the number of studies reporting pain improvement and their follow-up ranges (weeks to 12 years) rather than a pooled patient-level estimate.

For postoperative pain, the distribution reflects only the studies that reported pain outcomes. WAL was applied in 7 studies (*n* = 796 patients), of which 6 reported pain reduction (*n* = 732).^20,24,25,29,30,32,36^ SAL was used in 5 studies (*n* = 494 patients), all of which reported meaningful postoperative reductions in pain.^21,22,27,28,34^ UAL was described in 2 studies (*n* = 188 patients), both reporting benefit.^33,35^ LAL was used in 1 study (*n* = 38 patients), which also demonstrated pain reduction.^31^ Across modalities, pain reduction was observed early after surgery and was maintained in medium- and long-term follow-up. As in the QoL analysis, these numbers differ from the overall technique counts because each outcome subgroup includes a different set of studies.

### Differences Between Water Jet–Assisted Liposuction and Tumescent Infiltration, and Cost Considerations

Across the included studies, WAL and tumescent infiltration were both used, but direct comparative data were limited. Cost information was sparsely reported and varied substantially between studies, preventing reliable numerical comparisons.

Patient-reported satisfaction was consistently higher in studies using WAL, and postoperative discomfort was described as lower compared with conventional techniques in the studies that reported this outcome.^15^ Five studies reported cannula size, most frequently using 2 to 4 mm instruments, with larger cannulas reserved for deep-plane liposuction in advanced cases.

A formal meta-analysis was not performed because of substantial heterogeneity across studies. Clinical heterogeneity was present because of differences in surgical techniques (SAL, WAL, UAL, LAL), infiltration strategies, and patient populations. Methodological heterogeneity was evident in the use of different pain scales, QoL instruments, and outcome definitions. Statistical heterogeneity could not be estimated because of incomplete reporting of variance measures. Accordingly, findings are presented descriptively with aggregated counts and percentages.

## DISCUSSION

Liposuction was effective in improving the quality of life of patients with lipedema and relieved pain, sensitivity to pressure, bruising, aesthetic changes, weight, and mobility difficulties, as also summarized in published guideline reviews.^39^ There are clinical studies that used tumescent infiltration in combination with various suction or energy-assisted modalities, however, tumescent infiltration is an adjunct (infiltration strategy) and not a suction modality itself, so its predominance in the literature does not establish comparative superiority.^19^ This systematic review discussed the various aspects of liposuction techniques and their results in treating lipedema.

Ten studies reported changes in weight, BMI, clothing size, and body fat of patients undergoing intervention.^18-20,22,24,27,31-33,35^ Wright et al observed decreased body fat (47.0% ± 7.5% to 43.0% ± 8.5%), also confirmed by computed tomography and by the median reduction in limb circumference that resulted in a proportional body at the end of the treatment course.^18,19,22,27,31^ Mean reductions of 6 to 9 cm were achieved in the thighs, 4 to 6 cm in the middle of the lower legs, and 3 cm in each arm.^20,22,33^ However, Baumgartner et al showed that after 12 years postoperatively, body weight increased by 0.5 kg, with weight gain in ∼43.3% of the patients.^23^ Peled et al also observed a weight gain of 9 kg at the 4-year follow-up; however, the patient remained satisfied with the improvement in leg contour, maintained despite the weight gain.^31^

Water jet–assisted liposuction (WAL) uses a pulsatile water jet to dislodge adipose tissue before aspiration and requires dedicated equipment, specific disposables, and trained personnel, leading to higher upfront costs. In contrast, tumescent infiltration relies on inexpensive anesthetic and vasoconstrictive solutions and can be used with multiple suction modalities, although large fluid volumes or staged sessions may increase total procedural time. Therefore, cost comparisons between techniques should distinguish capital/equipment costs from per-session consumable and perioperative costs, and studies should report both whenever possible.^10,14,19^ Surgeon experience also strongly influences outcomes, with more experienced surgeons demonstrating lower complication rates and superior aesthetic results. Wright et al highlight that technical choice may depend on specific surgeon training, whereas Schlosshauer et al found high patient satisfaction with WAL because of reduced postoperative discomfort.^15,18^ Some experts recommend using blunt cannulas 2 to 4 mm in diameter to minimize the theoretical risk of lymphatic injury, reserving larger cannulas (>4 mm) for deep-plane liposuction in advanced cases, however, evidence supporting this recommendation remains limited and is largely based on expert consensus rather than high-quality comparative data.^22,25,27,28^

Of the 25 studies, 15 reported reduced pain complaint scores among patients with lipedema after liposuction compared with preoperative values. In Wollina and Heinig, the median pain score on a 10-point visual analog scale decreased from 7.8 points before liposuction to 2.2 after the treatment course.^20^ Dadras et al reported that the mean spontaneous pain score significantly reduced, with a mean difference of 3.5 points (*P* < .001).^21^ Similarly, the mean spontaneous pain score decreased significantly in preoperative studies by Schmeller et al, Baumgartner et al, and Rapprich et al, all showing a significant reduction (*P* < .001).^22,23,27^

Studies also reported improvement in pain and pressure sensitivity, feeling of tension, heavy legs, bruising, cosmetic impairment, walking limitations, and movement restriction.^14,20-23,27,28,33,35,36^ Liposuction improved knee mechanics, range of motion, and gait, which decreased pain and improved walking speed, stair climbing, sitting, and lifting functions.^18^ Herbst et al found that ambulation improved more in stage 3 lipedema (96.0%).^19^

The improvement in quality of life in patients undergoing liposuction is a significant parameter in the results of the treatment course. The best indices of quality of life were found in social and daily life, followed by physical complaints areas.^15^ In addition, patients had gains in emotional and social well-being, satisfaction with the appearance of the limbs, and the subjective quality of sexual life.^14,18,29,36^ Wright et al found significant improvement in all 8 domains of the SF-36 questionnaire.^18^ A study showed that the number of liposuction sessions performed and the general health status were correlated, suggesting that a greater number of liposuction treatment sessions has a positive effect on the general health status of patients with lipedema.^15^

Some studies showed that liposuction does not decrease lymphatic function in patients with lipedema, analyzed by lymphoscintigraphy months after the treatment course.^16,31,37^ Positive and lasting results may be guaranteed when the technique focuses on preserving the lymphatic system.^35^ Van de Pas et al showed slight improvements in mean clearance and mean inguinal uptake after tumescent liposuction.^16^ Therefore, liposuction can be considered an efficient and safe treatment for lipedema and may also improve lymphatic drainage in patients with this condition.^16,37^

Immunohistological analyses revealed the presence of a few lymphatic vessel structures in lipoaspirates. An analysis of liposuction aspirates from 60 lower limbs obtained from the inner knee region, a high-risk area for this type of treatment course, showed only minimal injury or no injury in the lymphatic vessels when the liposuction treatment course was performed strictly parallel to the axis of the lymphatic collectors.^25^ The immunohistochemical evaluation also confirmed that a tumescent infiltration state is not required for the WAL treatment course, preserving the structure of the lymphatic vessels and allowing for the initiation of the treatment course immediately after the effect of anesthesia. However, larger studies are needed to confirm these findings.^26^

In addition, the low rates of complications caused by liposuction reveal its safety for treating lipedema.^36^ Patients were absent from work for a mean of 2.7 weeks postoperatively, however, all complications were treated conservatively, with rest, elevation of the limbs, use of compression garments, and application of heparin.^24^ The side effects completely disappeared within a few weeks.^29,34^ In studies reporting serious adverse events, the infection rate ranged from 0.0% to 1.4%, and the bleeding rate was ∼0.3% after the treatment course.^20,22^ Nonetheless, none of these patients had a recurrence of lipedema, suggesting a long-term benefit.^20,24^ Heparin was used for prophylaxis against deep vein thrombosis (DVT), not as treatment.

The improvements caused by liposuction remained during the follow-up period.^30,36^ Those benefits resulted in tissue changes consistent with stage regression in some patients, such as the absence of lobules in patients with stage 3 lipedema.^18^ Baumgartner et al showed maintenance in the improvement of spontaneous pain, pressure sensitivity, edema, and movement restriction 4 years after the treatment course.^30^ Similarly, beneficial results were noted in the self-assessment of patients regarding aesthetic appearance, quality of life, and general impairment.

One study observed that better and long-term results could be achieved if patients were treated in the early stages of lipedema.^19,21^ However, UK guidelines suggest liposuction after 6 to 12 months of adherence to conservative therapy, and Dutch guidelines suggest liposuction for patients who no longer respond to conservative therapy.^19^

In some studies, the need for conservative therapy was significantly reduced and could be withdrawn (5.3% of patients) or continued to a lesser extent (23.4% of patients) after the liposuction.^21,22,24,28,35^ After 12 years of postoperative, Baumgartner et al showed that 54.0% of patients still underwent manual lymphatic drainage and wore compressive garments.^23^ Only 19.0% of patients required fewer conservative treatments than before, and 27.0% no longer required lymphatic drainage or compression therapy.

Liposuction is promising as a treatment for lipedema, especially in patients who do not respond to conservative treatments. However, standardized liposuction protocols and clarity on the role of tumescent infiltration are urgently needed. Specific recommendations for standardization may include defining the composition of the tumescent solution, guidelines for volume and infiltration rate, the precise time and duration for tumescent infiltration, and improving patient selection criteria. In addition, outlining surgical techniques, postoperative care strategies, and outcome measurement protocols may significantly increase the efficacy and safety of liposuction.

Direct head-to-head comparisons between suction/energy modalities are scarce. Many included studies reported favorable outcomes using tumescent infiltration in combination with various suction techniques, a smaller subset reported outcomes after WAL or energy-assisted techniques. Where comparative data were available, some series suggested that WAL or energy-assisted methods may be associated with reduced immediate bruising or faster early recovery, but these findings derive mainly from nonrandomized cohorts or case series and varied in outcome definitions. Because tumescent infiltration is not a suction modality but an infiltration strategy used across techniques, the predominance of studies using tumescent does not establish its superiority. Rigorous randomized or otherwise well-controlled comparative studies are required to draw firm conclusions.

This review has several important limitations. First, the body of evidence is dominated by nonrandomized observational studies (case series, retrospective cohorts, and a number of prospective cohorts), with no randomized controlled trials directly comparing suction modalities or infiltration strategies, this limits causal inference. Second, there was substantial heterogeneity in surgical technique reporting (type of suction device, cannula size, infiltration composition, and volumes), perioperative care (DVT prophylaxis, antibiotics, compression protocols), and outcome measurement (different pain scales, disparate QoL instruments, and variable follow-up durations), which prevented formal quantitative pooling. Third, many primary studies did not report outcome-specific denominators or detailed adverse event reporting, limiting our ability to compute precise event rates for some outcomes. Fourth, surgeon experience, center volume, and patient selection criteria were inconsistently reported and likely contributed to outcome variability. Finally, publication and reporting biases cannot be excluded. These limitations underline the urgent need for standardized reporting (including the composition of tumescent solutions, device settings, cannula diameters, and outcome definitions) and for prospective controlled studies.

## CONCLUSIONS

Liposuction was associated with symptomatic improvement and low rates of serious complications across included observational series, with many patients reporting reduced pain and improved quality of life. The majority of studies used tumescent infiltration; however, because high-quality head-to-head comparisons are lacking and tumescent infiltration is an adjunct rather than a suction modality, no definitive conclusion on superiority of tumescent vs nontumescent approaches can be drawn. Standardized reporting and prospective comparative studies are required to determine optimal technique, infiltration composition, and postoperative protocols.

## Supplemental Material

This article contains supplemental material located online at https://doi.org/10.1093/asjof/ojag039.

## Supplementary Material

ojag039_Supplementary_Data
