# Supplementary material for: Safety and Efficacy of Surgical Techniques in Treating Lipedema: Systematic Review
Source: Aesthet Surg J Open Forum. 2026 Feb 24;8:ojag039. doi: 10.1093/asjof/ojag039 (PMC13010320; doi:10.1093/asjof/ojag039)
Supplement: ojag039_Supplementary_Data [file ojag039_supplementary_data.zip › Supplemental Table 1.docx]

**Supplemental Table 1**. Characteristics of the Included Studies

| Author/year of Publication | Country | Study Design and Sample Size | Patient Characteristics | Liposuction Techniques | Intervention Characteristics | Follow-up | Results |
| --- | --- | --- | --- | --- | --- | --- | --- |
| KIRSTEIN et al., 2020^14^ | Germany | Retrospective study with 56 patients. | Mean age of 40.72 (± 12.54) years.  Mean body weight: initial 96.16 kg (± 23.11) and 93.69 kg (± 19.90) after the procedure.  The mean body mass index (BMI) was 33.13 (± 7.80) kg/m^2^ and 32.61 (± 6.69) kg/m^2^ after the procedure.  33.60% of patients before the procedure had stage three lipedema. | Not described. | A mean of 3831.00 (± 1971.08) mL of fat was aspirated per procedure and patient.  30.36% of patients had one, 28.57% had two, 28.57% had three, and only 12.50% had four or more procedures.  Procedures occurred in lower legs (57.00%), upper legs (23.00%), arms (11.00%), and buttocks (9.00%). | NR | Improvements were observed in pain, sensitivity to pain and pressure, tension, heavy legs, walking limitations, QoL, satisfaction with the appearance of the extremities, mental state, and occupational disability. |
| SCHLOSSHAUER et al., 2021^15^ | Germany | The first part of the study was a retrospective analysis, and the second was a prospective pilot study. The sample comprised 69 female patients. | The mean age was 50.6 ± 12.8 years at the time of the procedure, and the mean BMI was 33.4 (± 7.1) kg/m^2^. | Tumescent and lymph-sparing liposuction on the lower limb | The mean number of liposuction sessions was 2.9 (± 1.9), with sessions needed varying from one or up to 12 sessions.  The mean volume of fat removed per side was 1868 (± 885.5) mL. | NR | After liposuction, patients with lipedema showed significant improvement in disease-specific QoL across all subscales and overall score (2.9 ± 1.0). The best QoL was in social and daily life areas (2.8 ± 1.1 each), followed by physical complaints (2.9 ± 1.0). |
| VAN DE PAS et al., 2020^16^ | Netherlands | Observational, retrospective study with 117 women with lipedema in the lower limbs. | The mean age was 40.9 years (21.0 to 64.1 years).  The lipedema diagnosis was established by clinical history and physical examination.  Lymphatic function was compared between patients with lipedema and healthy individuals. | Tumescent liposuction. | The technique is described by Klein et al. (1990). | NR | The mean clearance of the feet was not significantly improved after tumescent liposuction (p = 0.37). Mean inguinal uptake slightly improved after the procedure (p = 0.02). |
| SEEFELDT et al., 2023^17^ | Germany | Retrospective cohort study with 860 patients with lipedema. | Almost all patients were women; only two were men.  The mean BMI was 32.00 (± 7.69) kg/m^2^.  A total of 124 patients were classified as stage 1 lipedema (14%), 523 patients as stage 2 (61%), 165 patients as stage 3 (19%), and 18 patients as stage 4 (2%); 30 patients (3%) did not respond to any stage. | Not described. | Not described. | NR | The pain was reduced after liposuction. The perception of lipedema between the stages and the perception of pain reduction was significantly different. In addition, regarding other comorbidities, menstrual complaints (43%), insomnia (36%), and migraine (35%) were mostly reported. |
| WRIGHT et al., 2023^18^ | USA | Case series of retrospective review that analyzed outpatient clinical records of 189 women with lipedema (507 procedures assessed for comorbidities, volumes, procedure time, and complications). | Mean age = 51.2 (± 11.3) years.  Mean BMI = 34.5 (± 7.5) kg/m^2^.  Extra comorbidities included arthritis (29.0%) and generalized joint hypermobility (50.5%), along with others. | Pre-surgical mapping of major superficial veins and lymphatic structures at the surgical sites (arms or legs) was conducted using vascular Doppler ultrasound or indocyanine green lymphography. Tumescent anesthesia was administered according to published guidelines, with lidocaine doses limited to 35–55 mg/kg per procedure. The risk of deep vein thrombosis (DVT) was assessed for each patient. | The procedure began with patients in the supine position, which was adjusted as needed to allow longitudinal access of the cannula around superficial venous and lymphatic structures, reducing the risk of vessel transection. Tumescent solution was infiltrated using a 16-gauge Monty tip cannula and Klein pump. The average procedure lasted 5.2 hours, with a mean total fat removal of 5.0 liters. The anterior thighs had the highest mean aspirate (7.0 liters), while the calves/ankles had the lowest (3.8 liters). | NR | The procedure improved knee mechanics, range of motion, gait, and reduced pain, while enhancing walking, stair climbing, and overall well-being. 68% of stage 3 lipedema patients saw regression, with a significant reduction in body fat percentage (47.0% to 43.0%). All SF-36 health domains improved. Complications included postoperative anemia and DVT, with 5.5% of patients experiencing dizziness from a hemoglobin drop. No other complications occurred. |
| HERBST et al., 2021^19^ | USA | Cross-sectional study with 148 female patients | Patients with stage 2 or 3 lipedema, were white, and aged about 50 years.  More than half reported hypermobile joints, corroborating previous data.  Except for those with stage 1 lipedema, all patients were obese (mean BMI > 30 mg/m^2^). | The tumescent technique (61.0%) or WAL (38.0%) were used. Only three patients had other procedures. | Patients had a mean of 2.4 (± 1.3) procedures; the maximum number was six.  Most procedures were performed by a plastic or cosmetic surgeon (63.0%), with the second largest group performed by dermatologists (13.5%) and the third by vascular surgeons (11.5%). | NR | QoL improved in 84.0%, and pain improved in 86.0% of patients. Ambulation improved most in stage three lipedema (96.0%). Weight loss occurred at all stages three months after the procedure. Areas of loss of fat included knees (44.0%), ankle (38.0%), inner knee area (37.0%), and forearm (23.0%). Dress size decreased by two sizes (n = 133), pants size decreased by three (n = 141), and shirt size decreased by one (n = 140). Complications included anemia, DVT, pulmonary embolism, lymphedema, pneumonia, cellulitis, skin irritation, folds of tissue or skin, asymmetry, wrinkles, or sagging of the skin. |
| WOLLINA; HEINIG, 2019^20^ | Germany | Retrospective cohort study with 111 patients. | All patients were female, aged between 20 and 81 years (44.0 ± 16.8 years). Among the patients assessed, seven patients presented stage 1 lipedema, 50 presented stage 2, and 48 presented stage 3. All patients had leg involvement in the upper (n = 108) or lower (n = 2) segments. Also, 80.0% of patients had at least one comorbidity. No pre-surgical glucose-6-phosphate deficiency was observed. | Cannular micro-liposuction in tumescent anesthesia  with classical mechanical liposuction; some patients underwent 980 nm diode LAL. | In 111 patients undergoing 334 procedures, a median of 4,700 mL (± 7,579) of fat was removed per session, ranging from 950 to 14,250 mL. Anesthesia used a 0.07% prilocaine solution with epinephrine in Ringer's solution, with a maximum of six liters per session, delivered at 40 mL/min. Liposuction was performed using 2 to 3-mm blunt cannulas and a vacuum pump with a negative pressure of 686 mmHg, without general anesthesia. Low-volume liposuction (< 4 L per session) was done in several sessions spaced 6-8 weeks apart. Small incisions were closed with polyamide stitches, and no drains or antibiotics were needed. Patients wore compression garments for at least six months. | 12 months | Thigh circumference reduced by 6.0 (±1.6) cm, and median pain levels decreased from 7.8 to 2.2 after treatment. Mobility improved for all patients, with 86% reporting improvements or full recovery. Bruising improved slightly by 20.9% and almost completely by 29.1%. Adverse events occurred in 1.2% of procedures, with a 0.0% infection rate and 0.3% bleeding rate. No lipedema recurrence was observed. In 4.5% of advanced cases, additional procedures like thigh lifts or laser lipolysis were performed for optimal results. |
| DADRAS et al., 2017^21^ | Germany | Longitudinal cohort study with 33 female patients. | The age ranged from 23 to 64 years (median of 45 years). BMI ranged from 24.5 to 50.6 kg/m^2^ (mean of 35.3 kg/m^2^). After treatment, BMI ranged from 22.7 to 47.2 kg/m^2^ (mean of 33.9 kg/m^2^).  All patients had lipedema in the lower limb. Nine patients presented additional upper limb involvement (36%). One patient had stage 1 lipedema, 11 patients had stage 2, and 13 patients had stage 3. | A total of 72 liposuction procedures were performed: 41 used UAL, and 31 used WAL. | Liposuction was performed with saline and epinephrine under general anesthesia in a hospital to enhance comfort and reduce operating time. Compression garments were worn immediately post-procedure, with new garments measured three weeks later. Manual lymphatic drainage (MLD) was allowed after the second postoperative day. The average operating time, including infiltration, was 116 minutes, with a mean fat removal of 3,106 mL per session and a total of 9,914 mL per patient. | 8 years | Pain, pressure sensitivity, feeling of tension, bruising, cosmetic impairment, and overall impairment of QoL significantly reduced from the preoperative period to the first postoperative follow-up; these results remained consistent until the second postoperative follow-up. The need for conservative therapy was significantly reduced.  Complications included erysipelas (n = 1), requiring treatment with antibiotics. However, no other complications were recorded, resulting in. a complication rate of 1.39%. |
| SCHMELLER, HUEPPE, MEIER-VOLLRATH, 2012^22^ | Germany | Observational study with 112 patients. | The mean age of patients was 38.8 years (20.0 to 68.0 years), and the mean weight was 79.3 kg (50.0 to 123.0 kg). A total of 35 patients had stage 1 lipedema, 75 had stage 2, and two had stage 3. Almost all patients underwent conservative therapy for many years and reported no improvements or progression of subcutaneous fat volume. | Liposuction under tumescent local anesthesia with UAL. | Liposuction was performed on the legs, hips, and arms. The mean amount of tumescence was 7,707 mL (2,564 to 13,450 mL), and the mean duration of procedures was two hours (40 minutes to 3 hours and 35 minutes). Of the 112 patients, 12 underwent one procedure, 29 underwent two procedures, 71 underwent three or more procedures. The minimum time between procedures was one month, and the maximum was one year.  The mean of fat removed was 9,846 mL per patient (1,000 to 25,600 mL) or 3,077 mL per session (450 to 7,000 mL), depending on the size and number of treated areas, with improved shape and normalization of body proportions. | 12 years | The reduction of subcutaneous fat decreased the circumference of the hips, legs, and arms, resulting in a proportional body after the procedure. The thighs (inguinal region) were reduced by 8 cm (1 to 23 cm) and 4 cm (1 to 11 cm) in the middle of the lower legs (calves). The mean weight before the procedure was 79.3 kg (50.0 to 123.0 kg) and 78.9 kg (49.5 to 118.0 kg) before the last liposuction.  Spontaneous pain, pressure sensitivity, edema, movement restriction, and aesthetic impairments significantly reduced or disappeared, increasing QoL. Patients with stage 2 and 3 lipedema showed more significant gains than those with stage 1. Decongestive physiotherapy was either continued (with less frequency) or could be omitted. No serious complications (wound infection rate of 1.4%, bleeding rate of 0.3%) were observed. |
| BAUMGARTNER et al., 2021^23^ | Austria | Longitudinal cohort study with 60 female patients. | The mean age was 54.1 years (35.0 to 80.0 years).  The mean weight before the first liposuction was 79.7 (50.0 to 116.0) kg.  The mean postoperative time interval was 13 years and three months after the first procedure and 12 years and four months after the last procedure.  Of the 60 patients, 18 (30%) had stage 1 lipedema, and 42 (70%) had stage 2 before liposuction.  No patients had stage 3 lipedema. | Tumescent liposuction. | The areas to be treated were identified and marked. Then, a clinical analysis was performed, as well as a discussion of expectations and possible complications. The tumescent solution (lidocaine, epinephrine, and saline) was injected into areas to be treated. | NR | Twelve years postoperatively, the average body weight change among patients was an increase of 0.5 kg, with one patient remaining at the same weight. A total of 33 patients (55.0%) lost weight, averaging 6.2 kg, while 26 (43.3%) gained weight, averaging 7.9 kg. The average age of those who lost weight was 34.2 years (postoperative weight 79.5 kg), and for those who gained, it was 43.5 years (postoperative weight 84.0 kg). Long-term improvements included reductions in spontaneous pain, pressure sensitivity, edema, bruising, and movement restrictions, alongside better self-assessed cosmetic appearance and quality of life. Although complaints slightly increased between four to eight years, they stabilized between eight and twelve years. Additionally, 20 patients (54.0%) continued manual lymphatic drainage (MLD) and wore compression garments, while 19.0% required fewer conservative treatments, and 27.0% no longer needed them. |
| WITTE et al., 2020^24^ | Germany | Retrospective cohort study with 63 patients | The mean age at the first procedure was 35 years, and the mean BMI was 28.4 (± 0.6) kg/m^2^. About 18 patients (29%) had stage 1 lipedema, and 45 (71%) had stage 2 lipedema. Throughout treatment, six patients (10%) underwent one, 21 (33%) two, 24 (38%) three, and 12 (19%) four procedures. | All procedures used a WAL device (body jet/Human Med AG, Schwerin, Germany) | Procedures were performed under tumescent anesthesia combined with conscious sedation, using a specific saline and anesthetic mixture. Cefazolin was given as prophylaxis. Liposuction utilized 3.5 mm and 4.8 mm cannulas, with varying infiltration volumes based on limb type. Patients were positioned differently for optimal fat removal, particularly in the lower and upper legs and buttocks. Incisions were closed with 5–0 Prolene stitches, with some left open for drainage. Postoperatively, patients received compression garments, low molecular weight heparin for seven days, and were monitored overnight. Compression protocols were gradually reduced, and MLD began two days post-op. On average, 12,922 mL of fat was removed per patient across procedures. | NR | All symptoms decreased significantly in severity at follow-up (22 months after the procedure). All patients wore compression garments and received MLD preoperatively, which reduced the need for conservative treatment to 44.0%.  Patients lost a mean of 5.6 kg compared with their preoperative weight, resulting in a mean BMI decrease of 2.3 kg/m^2^.  No significant complications occurred. Postoperative edema occurred for a mean of 4.3 weeks, and patients were absent from work for a mean of 2.7 weeks. No patient presented a recurrence of lipedema in the follow-up period. |
| STUTZ; KRAHL, 2009^25^ | Germany | A case study with histological and immunohistochemical analysis of 30 female patients. | Patients were aged between 21 and 63 years and had pre-existing pronounced lipedema. | WAL (BODY-JET system, human med AG, Schwerin, Germany) in both legs under standardized conditions with reduced amounts of Klein solution (1.0 to 1.5 liters). | Infiltration was performed in all cases using a body-jet infiltration cannula (3.5 mm diameter) until sufficient anesthesia was obtained with the prepared solution. The liposuction started without waiting for fluid infiltration. For all procedures, the irrigation-aspiration cannula (3.5 mm) was directed strictly along the axis of the lymphatic collectors. Both legs received the same treatment. The vacuum was adjusted to a constant of 0.6 to 0.8 bar.  The amount of aspirated fat ranged from 250 to 2,350 ml. | NR | Minimal injury or no injury was caused to the lymphatic vessels if the liposuction was performed strictly parallel to the axis of the lymph collectors; such analysis occurred according to the aspirates from 60 lower limbs obtained in the internal region of the knee, a high-risk region for this type of operation. Histological analysis of aspirates showed that most lipocytes were intact and presented low vascular quantity. The immunohistochemical evaluation also confirmed that WAL does not need a tumescence state since it preserves the lymphatic vessels. Thus, a paradigm shift occurred with the introduction of WAL. Similarly, no pre-infiltration period is required for homogenization of fat. |
| WRIGHT; HERBST, 2022^26^ | USA | Case series with three women. | Three women with lipedema who had no previous venous or lymphatic disease developed recent-onset symptomatic lymphedema (stage 2 or 3) and skin and tissue changes within six months to one year after suction lipectomy for the treatment of lipedema in the legs. Each of the three women had their procedures performed using different suction devices and under different types of anesthesia. Two of the lymphatic injury cases had subsequent nuclear lymphoscintigrams that confirmed impaired lymphatic function. | Each woman performed the procedure using WAL, EAL, and UAL procedures. | Not described. | NR | Blunt suction lipectomy using a dry technique can harm lymphatic collectors. Tumescent anesthesia mitigates tissue damage, with the longitudinal technique showing no injury or only moderate injury, while the cross-sectional technique had no severe injury. However, tumescent anesthesia alone did not prevent reported complications. One case involved lymphatic and dermal issues from ultrasound-assisted liposuction (UAL), with heat potentially increasing injury risk. Guidelines in the UK, Germany, the Netherlands, and Spain advise against using additional energy during liposuction. Two cases involved general and tumescent anesthesia, which may mask inadequate tumescent coverage and elevate injury risk; Dutch guidelines specifically advise against general anesthesia for lipedema treatment. |
| RAPPRICH; DINGLER; PODDA, 2011^27^ | Germany | Prospective cohort study with 25 patients. | Patients were aged between 22.0 and 65.0 years (mean 38.0 ± 12.5 years). Lipedema affected the entire leg (n = 20), the thigh (n = 3), and the lower leg (n = 2). | Liposuction under tumescent local anesthesia with 4 mm diameter vibrating cannulas and handpiece (VibraSat ®, Möller Medical, Fulda). | The tumescence solution used for liposuction included prilocaine, suprarenin, sodium bicarbonate, triamcinolone, and 0.9% sodium chloride, with prilocaine concentration around 0.05%. Patients typically received about 6,000 mL of solution per session, averaging 5,155 ± 1,304 mL, administered via a roller pump. Infiltration rates varied based on region and pain tolerance, generally between 120 and 200 mL per minute, with sedation provided as needed. For aspiration, vibrating cannulas with three blunt openings were employed. The average aspirated volume was 2,482 ± 968 mL, with 1,909 ± 874 mL being pure fat. Postoperatively, patients received antibiotic and DVT prophylaxis, with compression garments applied for the first seven days, followed by daytime use for four to six weeks. MLD was initiated two to three times per week for at least six weeks. | NR | Leg volume was reduced by 6.9%. Pain significantly reduced from 7.2 (± 2.2) to 2.1 (± 2.1) (p < 0.001). Pressure sensitivity and bruising also significantly improved. QoL (psychological distress caused by lipedema) improved from 8.7 (± 1.7) to 3.6 (± 2.5) (p < 0.001).  Other parameters also significantly improved, and the overall severity score improved in all patients.  Despite the preventive measures, one patient presented DVT in the leg one week after the procedure. The condition was treated promptly without further complications.  The measured volume reduction and symptom improvement were not correlated.  About 15 of the 25 patients (60.0%) received MLD before liposuction. Two patients (8.0%) were also treated after the procedure, and 19 patients reported regular use of compression therapy before liposuction (76%). |
| SCHMELLER; MEIER-VOLLRATH, 2006^28^ | Germany | Case series with 21 patients. | Lipedema stages 1 and 2. The mean age was 37.7 (22.0 to 63.0) years. All underwent conservative therapy for many years. | Liposuction under tumescent local anesthesia with vibrating microcannulas. | Liposuction was performed on the deep subcutaneous fat on the thighs (inner, outer, and frontal sides) and circumferentially on the deep and superficial fat of the calves.  The mean amount of tumescent solution infiltrated was 7,881 (2,740 to 13,500) mL, the infiltration time ranged between 1.0 and 1.5 hours, and the duration of the procedure ranged between 1.0 and 2.5 hours.  The mean amount of fat removed per session was 3,017 (1,060 to 5,500) mL, depending on the size and number of treated areas. | 8 years | All patients presented great improvement; body proportions were normalized. In addition, spontaneous pain, pressure sensitivity, and bruising disappeared completely or improved markedly.  All patients reported increased QoL. Physiotherapy continued to a lower degree.  Other than minor edema for a few days, no complications occurred. |
| GHODS et al., 2020^29^ | Germany | Retrospective analysis of 106 patients who underwent 298 liposuctions. | A total of 77 patients (73.0%) reported a positive family history of lipedema.  Most patients reported disease onset during puberty (n = 62) or pregnancy (n = 22). The prevalence of comorbidities in 93 patients (88.0%) indicated at least one concomitant disease. | EAL and WAL. | All patients received conservative preoperative therapy for at least six months. Liposuction was performed under general anesthesia at the hospital. The procedure was performed using a "wet technique" with infiltration of a maximum of six liters of tumescent solution (1ml of adrenaline 1:1,000 in 1,000ml of NaCl) to protect the lymphatic system. The mean volume of liposuction per procedure was 6,355 (±2,797) mL, with a mean total fat aspirate volume per patient of 17,887 (±10,341) mL throughout the treatment. | NR | The procedure significantly reduced pain perception. About 58.0% of patients reported needing less or no disease attestation due to lipedema-related symptoms after the procedure. Subjective quality of sex life significantly improved. All patients would recommend multi-stage surgical therapy according to a median global satisfaction of 90 points (from 0 to 100).  The complication rate was low, with four superficial wound infections (1.3%), two seromas (0.7%), and one case of mild postoperative bleeding (0.3%) not requiring blood transfusion. All complications could be treated conservatively. |
| BAUMGARTNER; HUEPPE; SCHMELLER, 2016^30^ | Austria | Long-term prospective cohort study with  85 patients. | The mean age was 40.1 years (22.0 to 68.0 years), and the severity of lipedema was stage 1 in 31% patients, stage 2 in 67% patients, and stage 3 in 2% patients. The second evaluation included 28% patients with stage 1 lipedema and 72% with stage 2 lipedema. | Tumescent liposuction. | Not described. | NR | Spontaneous pain, pressure sensitivity, edema, and movement restrictions improved after four years of treatment. The aesthetic appearance, QoL, and overall impairment (self-assessments) also improved. Eight years after the procedure, the need for conservative treatment was similar to four years earlier. |
| PELED; SLAVIN; BRORSON, 2012^31^ | USA/Sweden | Case study with one patient. | A 21-year-old woman presented with bilateral chronic edema in her lower limbs, which had developed gradually over more than ten years. Initially thought to be lymphedema, lymphoscintigraphy ruled out that diagnosis. On examination, she was lean (68 kg, 173 cm, BMI 22.7) and exhibited significant edema from the inguinal region to the ankles, with a distinctive ring-shaped deformity at the ankle where the edema abruptly ceased. | Modified SAL in the lower limbs. | In the operating room, the patient's lower limbs were sterilized and tourniquets applied to reduce blood loss. After exsanguination with an Esmarch bandage, low molecular weight heparin was given for DVT prophylaxis. Approximately 15 small incisions (3 mm) were made for suction-assisted lipectomy (SAL) using MicroAire cannulas. The removed tissue was soft and free of fluid accumulation or fibrosis. Liposuction focused on the ankle to knee area, removing 1,400 mL of pure fatty oil and fat clusters, resulting in immediate limb contour improvement. Wounds were closed with Vicryl stitches, and bilateral compression garments were applied from the ankles to below the knees. The postoperative recovery was uneventful, with garments worn continuously for six months before being discontinued. | NR | After one year, the limb contour improved. A new lymphoscintigraphy performed two years after the procedure showed no change in the lymphatic system. Computed tomography, performed in the same period, showed a marked reduction of subcutaneous fat in the distal part of the lower limbs compared with preoperative images. At the 4-year follow-up, the patient gained 9 kg; however, she is still pleased with the improved limb contour, which has been maintained despite the weight gain. |
| WOLLINA; HEINIG; NOWAK, 2014^32^ | Germany | Case report of three patients | The women were 55, 72, and 77 years old, and their BMI was 33, 35, and 42 kg/m^2^, respectively. They presented advanced lipedema (legs) and multiple comorbidities. | LAL of the upper legs and knees, performed under tumescent anesthesia. Medial elevation of the thigh and partial lower abdominoplasty with minimal detachment to correct sagging skin and prevent intertrigo. Post-surgical care with non-elastic flat mesh compression straps and MLD. | The procedure focused on removing excess fat, with a maximum of five liters of tumescent solution used per session over four to five sessions. A 980 nm diode laser integrated into the liposuction cannula was utilized. Patients with advanced lipedema often had excess vertical tissue due to poor skin and fat adherence, necessitating a thigh lift for aesthetic improvement. This lift was performed at least four months post-liposuction under general anesthesia, with incisions marked preoperatively. Skin and fat were removed in anteroposterior fashion, using bipolar diathermy for hemostasis. Minimal detachment was done, protecting the greater saphenous vein. Wounds were closed in layers to reduce tension, and compression straps were worn for six months. In total, four to six liters of fat were removed. | NR | All patients developed methemoglobinemia within 12 hours post-liposuction, treated successfully with intravenous toluidine blue and resolved within 20 minutes. Each patient wore non-elastic flat mesh compression garments for six months and began mobilization on the day of the procedure. MLD started after two weeks. The procedures were well tolerated, with no major adverse events, though bruising was noted in all cases. A compression strap (46 mmHg) was recommended for the first two months, then for at least eight hours daily for four months. Moisturizers were advised for skin care. While BMI decreased slightly, body weight loss ranged from 5 to 10 kg, and clothing sizes reduced by two to three sizes. No secondary lymphedema or lipedema was observed during follow-up (two to four years), and patients expressed satisfaction with the results while continuing to wear compression garments during the day. |
| NAVADEH, 2019^33^ | Iran | Case series with 21 patients. | The mean age of the patients was 41 years (21 to 62 years), and the mean body weight was 86 kg (57 to 115 kg). Seven patients were in stage 1, nine patients in stage 2, and five patients reached stage 3 lipedema. | Liposuction under tumescent local anesthesia. | Liposuction was performed on trochanters, inner thighs, gluteal folds, and front thighs for stage 1 patients, while stages 2 and 3 also included arms and legs. All patients received prophylactic antibiotics (amoxicillin or erythromycin) for three days starting on the procedure day. Stage 1 patients underwent one session, while stages 2 and 3 required two or three sessions. The mean volume of tumescent fluid used was 4,750 mL per session. The BodyTite system, utilizing radiofrequency, was applied for 30 minutes before liposuction to prevent skin sagging. Liposuction was conducted using the Power-Assisted Lipoaspiration System (PAL-650), employing a 4-mm diameter cannula to minimize skin irregularities. The average operative time was 2 hours and 50 minutes, with a mean fat removal of 2,250 mL per session. Patient evaluations took place at six weeks, three months, six months, and one-year post-procedure. | NR | Patients had cosmetic results and improved QoL after liposuction. Pain, skin hypersensitivity, ecchymosis, and edema decreased in all patients after the procedure. The reduced subcutaneous fat from the lower and upper limbs improved the body contour. The mean size reduction of each thigh was 9 cm (4 to 14 cm), of each calf 4 cm (2 to 6 cm), and of each arm 3 cm (2 to 4 cm).  The patients had no major complications. Inflammation of the incisions occurred in four patients, and three patients suffered from orthostatic reactions the night after treatment. Ecchymosis, especially in the inner thighs and knees, occurred in eleven patients and disappeared within three weeks. One patient presented a hypertrophic incision scar. Also, one patient presented a circumscribed accumulation of fluid in the upper right leg during eight weeks after liposuction. The resulting trochanter irregularity in two patients was successfully corrected by lipofilling. |
| RAPPRICH et al., 2015^34^ | Germany | Cross-sectional study with  85 patients. | About 30% of patients had a normal weight (BMI from 20.0 to 25.0 kg/m^2^), 26% were pre-obese (BMI from 25 to 30.0 kg/m^2^), and 20% were obese (BMI over 30.0 kg/m^2^).  The entire leg was the most common location (62.4%), followed by the thigh (30.6%) and the calf (6.0%). | EAL | The tumescence solution was infiltrated using the Sattler method with a continuous roller pump until the skin became hard and elastic, achieving a "bleaching effect" from tissue pressure and adrenaline. Infiltration rates were adjusted based on patient pain, typically between 120 and 200 mL/min. After sufficient contact time, subcutaneous fat was aspirated longitudinally to protect the lymphatic system using a 4 mm blunt vibrating micro-cannula. Residual fluid was removed via massage. Postoperatively, patients received antibiotics (ciprofloxacin or cefuroxime) for three days and enoxaparin for thrombosis prophylaxis, with compression stockings applied continuously for the first week and during the day for four to six weeks. MLD was performed two to three times per week for at least six weeks, with treatment requiring a minimum of three sessions for the legs. | NR | Liposuction significantly reduced pain, bruising, and the tendency to swell in the limbs of all patients, improving their QoL.  About 42.9% of patients were treated with three sessions.  Postoperative hematomas occurred in 12 patients in 168 procedures (7.1%). Five of these patients presented mild hematoma, four patients had moderate hematoma, and for two patients, the hematoma required revision. After one liposuction, a seroma that required treatment occurred, and one patient presented thrombophlebitis.  The tired legs and sensitivity to touch were the main symptoms of lipedema that best responded to liposuction, with a mean of up to 4.7 points. The tendency to bruise also improved (mean of 3.8 points). |
| MÜNCH, 2017^35^ | Germany | Observational comparison with 141 patients with lipedema who were treated surgically; of these, 71 patients were reassessed at follow-up (median of 35.9 months). | The mean age was 37.2 years (18.0 to 65.0 years), the mean preoperative body weight was 72.5 kg (47.0 to 136.0 kg), and the mean BMI was 26.6 kg/m^2^ (18.6 to 44.9 kg/m^2^). | WAL. | All procedures were performed on an outpatient basis under local anesthesia and preoperative sedation with midazolam (7.5 mg), supplemented with nitrous oxide/oxygen if necessary. Prophylactic antibiotics (ciprofloxacin, 2 x 500 mg) were administered for three days, along with thrombosis prophylaxis based on risk profile. A 2.5 mm infiltration cannula was used to inject Klein's solution, preheated to 37°C, into the subcutaneous fat layer through small incisions. The solution was distributed fan-shaped at a flow rate of 188 mL/min, with a penetration depth of 40 to 50 mm. Following infiltration, liposuction was performed using a 30 cm double-lumen cannula, with negative pressure controlled by the operator. Incision sites were left open to drain excess fluid and closed spontaneously within 24 hours. The average procedure duration was 1 hour and 36 minutes, with an average fat removal of 4,200 mL per patient. Postoperatively, a compression garment was applied, suction zones were cooled to 16°C, and local heparin gel and paracetamol were prescribed for pain management. Compression garments were advised for continuous wear for 24 hours, then for two weeks, and during the day for an additional six weeks. | NR | The subcutaneous fat layer was significantly reduced, improving the proportions in all patients. In addition, all symptoms were reduced, and the QoL increased after a few weeks. Body weight was reduced by 3.4 kg (mean) after the procedure. Also, 42.1% of patients reduced the size of their clothes by one size and 10.5% by two sizes. Lymphatic drainage or compression treatments were no longer needed in 5.3% of patients, could be reduced by 23.4%, and were considered more effective in 9.5%  The anesthetic solution was selectively administered to the area to be aspirated, causing optimal analgesia and vasoconstriction. In addition, patients had less swelling, and liposuction was more accurate.  Overt bruising, swelling, and local hardening occurred in most cases; however, these side effects completely disappeared within a few weeks with rest, elevation, use of compression garments, and application of heparin preparations. |
| SANDHOFER et al. 2121^36^ | Austria | Retrospective data analysis of 27 patients (40 liposuction procedures). | The mean age was 41.7 years, the mean body weight was 90.3 kg, and the mean height was 167.4 cm, resulting in a mean BMI of 32.3 kg/m^2^. | Liposuction was performed under tumescent anesthesia using EAL. | The mean procedure time was 118 minutes, with an average of 6,111 mL of fat aspirated (mean of 5,585 mL). The mean anesthetic fluid used during tumescent anesthesia was 11,404 mL, delivering 3,061.9 mg of lidocaine (34.23 mg/kg) and 9.7 mg of epinephrine (0.11 mg/kg). Each patient typically required two or three leg procedures and one arm procedure, performed under tumescent anesthesia and superficial conscious sedation. Premedication included oral midazolam (7.5 mg), followed by intravenous midazolam (3.0 to 5.0 mg) and low-dose remifentanil. Anxiety management involved additional midazolam or clonidine. The tumescent fluid was preheated to 37°C and injected into subcutaneous fat using a surgical infusion pump by two personnel until the tissue achieved a firm turgor. After a 30-minute wait, liposuction commenced using the MicroAire pal Liposuction System with cannulas of 3 to 4 mm diameter, taking care to consider lymphatic vessel positioning. Intraoperative pain was addressed with minimal secondary infiltration if necessary. | NR | Liposuction was effective in reducing symptoms and fat associated with lipedema. Patients reported improvements in the appearance of the affected areas, and pain and discomfort were reduced.  Many patients improved their QoL after the procedure, with reduced lipedema-related symptoms and improved mobility and overall comfort. Beneficial results were sustained during the follow-up period.  No relevant complications associated with drug side effects, hypovolemia or hypervolemia, or blood loss were detected. |
| VITORASSO et al., 2023^37^ | Brazil | Case study of one patient. | A 34-year-old female patient presented painful enlargement of the arms and lower limbs caused by lipedema. The patient underwent conservative treatment with a slight improvement in pain and weight. Lymphoscintigraphy showed slow progression of radiotracing in the left lower limb, collateral, and tortuous lymphatic vessels in the right lower limb, and exuberant concentration of the radiopharmaceutical in the inguinal chain. | SAL of the arms and lower limbs. The level of liposuction was deep subcutaneous to preserve lymphatic vessels and avoid superficial irregularities. | Fat aspirate (3,900 mL) was taken from each lower limb with minimal bleeding (40 mL of blood pelleted in the collector). The 980nm subdermal LASER diode applied after liposuction promoted skin tightening on the thighs. In addition, bilateral arm elevation was performed to treat sagging skin. Low molecular weight heparin was administered perioperatively for DVT prophylaxis. Postoperative pain intensity was classified as seven according to the numerical scale until the fourth day after the procedure. Subsequently, the patient rated the discomfort as three and gradually disappeared by the third week after the procedure. The discomfort and edema improved with analgesics (dipyrone, profenide, and oxycodone) and lymphatic drainage. Compressive garments and bandages were applied every 24 hours for the first month after the procedure. Subsequently, the patient used compressive belts for half the day until the third postoperative month and intermittently until the sixth postoperative month, after which she stopped using compressive belts. | NR | The patient reported improvement in pain, discomfort, and heaviness in the legs, and satisfaction with the appearance of the limbs. A second lymphoscintigraphy was performed nine months after the procedure: lymphatic changes, normal radiopharmaceutical progression time, resolution of l, and symmetrical drainage pattern. Collateral lymphatic pathways and tortuous vessels were no longer identified. Thus, the treatment was efficient and safe for lipedema and might also improve lymphatic drainage in patients with this condition. |
| WOLLINA; GOLDMAN; HEINIG, 2010^38^ | Germany | Case series with six patients. | All patients were female and obese with a BMI of 34.0 to 41.9 kg/m2 (mean 38.2 ± 3.8 kg/m2) and ages ranging from 29 to 78 years (mean 55.7 ± 20.5 years); five of them had comorbidities. | Microcanular tumescent liposuction. | During liposuction, fat removal per session ranged from 500 mL to 1,800 mL, totaling 1,500 to 4,800 mL across all sessions, with no adverse events reported. All patients received a single preoperative intravenous dose of ascorbic acid to prevent methemoglobin formation; only one patient with a pacemaker received prophylactic antibiotics. Tumescence anesthesia was administered using a 0.1% prilocarpine solution, with a maximum of five liters used. Liposuction was performed with blunt cannulas (2-5 mm), and incisions were partially closed with polyamide sutures, eliminating the need for drainage. Compressive bandages were applied for 24-48 hours, followed by custom compression garments. Tissue collected during the procedure underwent histological analysis. Liposuction was conducted in one to four sessions to manage swelling, and postoperative blood counts and methemoglobin levels were monitored, treating methemoglobinemia with intravenous ascorbic acid until levels normalized. | NR | All patients tolerated the liposuction. Histology revealed large lobes of fat.  Although BMI did not change significantly, compression pants were used to decrease leg volume during sequential procedures.  The removal of large fat implies a better result for pain. Patient satisfaction was “high” or “very high”. Methaemoglobulinemia was the most common adverse effect registered. |

BMI: body mass index; QOL: quality of life; SF-36: 36-Item Short Form Survey; DVT: deep vein thrombosis; WAL: water-assisted liposuction; MLD: manual lymphatic drainage; SAL: suction-assisted liposuction; LAL: laser-assisted liposuction; UAL: ultrassound-assisted liposuction; EAL: energy-assisted liposuction
