# Supplementary material for: Safety and Efficacy of Surgical Techniques in Treating Lipedema: Systematic Review
Source: Aesthet Surg J Open Forum. 2026 Feb 24;8:ojag039. doi: 10.1093/asjof/ojag039 (PMC13010320; doi:10.1093/asjof/ojag039)
Supplement: ojag039_Supplementary_Data [file ojag039_supplementary_data.zip › Supplemental Table 2.docx]

**Supplemental Table 2**. Quality Assessment of Included Studies Using the Newcastle-Ottawa Scale (NOS)

| Study | Selection (0–4) | Comparability (0–2) | Outcome/Exposure (0–3) | Total Score (0–9) | Risk Category |
| --- | --- | --- | --- | --- | --- |
| Kirstein et al., 2020^14^ | 3 | 1 | 2 | 6 | Moderate |
| Schlosshauer et al., 2021^15^ | 3 | 2 | 2 | 7 | Low |
| Van De Pas et al., 2020^16^ | 2 | 1 | 2 | 5 | Moderate |
| Seefeldt et al., 2023^17^ | 3 | 1 | 3 | 7 | Low |
| Wright et al., 2023^18^ | 4 | 2 | 3 | 9 | Low |
| Herbst et al., 2021^19^ | 3 | 2 | 3 | 8 | Low |
| Wollina; Heinig, 2019^20^ | 3 | 1 | 2 | 6 | Moderate |
| Dadras et al., 2017^21^ | 2 | 1 | 2 | 5 | Moderate |
| Schmeller, Hueppe, Meier-Vollrath, 2012^22^ | 3 | 1 | 3 | 9 | Low |
| Baumgartner et al., 2021^23^ | 4 | 2 | 3 | 7 | Low |
| Witte et al., 2020^24^ | 3 | 1 | 2 | 5 | Moderate |
| Stutz; Krahl, 2009^25^ | 2 | 1 | 2 | 5 | Moderate |
| Wright; Herbst, 2022^26^ | 2 | 1 | 2 | 5 | Moderate |
| Rapprich; Dingler; Podda, 2011^27^ | 3 | 1 | 3 | 7 | Low |
| Schmeller; Meier-Vollrath, 2006^28^ | 3 | 1 | 3 | 7 | Low |
| Ghods et al., 2020^29^ | 3 | 1 | 3 | 7 | Low |
| Baumgartner; Hueppe; Schmeller, 2016^30^ | 4 | 2 | 3 | 9 | Low |
| Peled; Slavin; Brorson, 2012^31^ | 3 | 1 | 3 | 7 | Low |
| Wollina; Heinig; Nowak, 2014^32^ | 3 | 2 | 2 | 7 | Low |
| Navadeh, 2019^33^ | 2 | 1 | 2 | 5 | Moderate |
| Rapprich et al., 2015^34^ | 3 | 1 | 3 | 7 | Low |
| Münch, 2017^35^ | 3 | 2 | 3 | 8 | Low |
| Sandhofer et al. 2121^36^ | 4 | 2 | 3 | 9 | Low |
| Vitorasso et al., 2023^37^ | 3 | 1 | 2 | 6 | Moderate |
| Wollina; Goldman; Heinig, 2010^38^ | 2 | 1 | 2 | 5 | Moderate |
