# Supplementary material for: Safety and Efficacy of Surgical Techniques in Treating Lipedema: Systematic Review
Source: Aesthet Surg J Open Forum. 2026 Feb 24;8:ojag039. doi: 10.1093/asjof/ojag039 (PMC13010320; doi:10.1093/asjof/ojag039)
Supplement: ojag039_Supplementary_Data [file ojag039_supplementary_data.zip › Appendix.docx]

**PRISMA 2020 Checklist – Lipedema Liposuction Systematic Review**

This checklist is auto-filled where information was available in the manuscript. Fields that require author confirmation are marked as 'Author to verify'.

| Section/Topic | Page | Checklist item | Location where item is reported |
| --- | --- | --- | --- |
| TITLE | 1 | Identify the report as a systematic review. | Title: “Safety and Efficacy of Surgical Techniques in Treating Lipedema: Systematic Review” |
| ABSTRACT | 1 | Provide a structured summary. | Abstract structured with Background/Objective/Methods/Results/Conclusion |
| INTRODUCTION – Rationale | 2 | Describe the rationale. | Introduction: explains the need to evaluate safety and efficacy of surgical techniques in lipedema |
| INTRODUCTION – Objectives | 4 | Provide an explicit statement of the objective(s). | Introduction: objective clearly stated as assessing safety and efficacy of liposuction in lipedema |
| METHODS – Eligibility criteria | 5 | Specify inclusion and exclusion criteria. | Methods: inclusion/exclusion criteria stated (human studies, patients with lipedema, surgical liposuction intervention) |
| METHODS – Information sources | 5 | All databases, registers, etc., with date last searched. | Methods – PubMed, Scopus, MEDLINE; searched until June 2024 |
| METHODS – Search strategy | 6 | Present full search strategies. | Methods: detailed search strategy with keywords and filters included in manuscript |
| METHODS – Selection process | 5-6 | Specify methods used to decide study eligibility. | Methods: two-step selection – title/abstract screening and full-text review; duplicates removed. |
| METHODS – Data collection process | 6 | Specify methods for data collection from reports. | Methods: data extraction performed independently by two reviewers. |
| METHODS – Data items | 6 | List and define all outcomes and other variables. | Methods: extracted data – author, year, country, sample size, sex, technique, aspirated volume, sessions, follow-up, outcomes, complications. |
| METHODS – Study risk of bias assessment | 6 | Specify methods to assess risk of bias. | Methods – Newcastle–Ottawa Scale (NOS) |
| METHODS – Effect measures | Not applicable (descriptive review) | Specify effect measures for each outcome. | Methods: effect measures not quantitative; descriptive review; complication rates reported when available |
| METHODS – Synthesis methods | 6 | Describe methods of synthesis. | Methods: narrative synthesis grouping by technique, complications, and follow-up |
| METHODS – Reporting bias assessment | 6 | Describe any methods used to assess risk of bias due to missing results. | Methods: risk of bias assessed qualitatively |
| METHODS – Certainty assessment | Not applicable | Describe methods used to assess certainty (e.g., GRADE). | Methods: not applicable; descriptive review without meta-analysis |
| RESULTS – Study selection | 7 | Describe results of search and selection process. | Results: “The search found 858 records (412 PubMed, 358 Scopus, 88 MEDLINE). After removal of 29 duplicates, 829 were screened…” |
| RESULTS – Excluded studies | 7 | Cite studies that appeared to meet criteria but were excluded, with reasons. | Figure 1: PRISMA flow diagram of included/excluded studies. |
| RESULTS – Study characteristics | 8-10 | Cite characteristics of included studies. | Table of study characteristics (Author, Country, Design, N, Technique, Sessions, Volume, Follow-up, Outcomes, Complications) |
| RESULTS – Risk of bias in studies | Supplementary Table | Present risk of bias assessments. | Table 1: NOS assessment for each study |
| RESULTS – Results of individual studies | 11-16 | Present summary statistics, effect estimates. | Table of included studies reporting main outcomes and complications |
| RESULTS – Results of syntheses | 12-16 | Present results of syntheses. | Text: summary of efficacy, safety, and complications grouped by technique |
| RESULTS – Reporting biases | NR | Present assessments of risk of bias due to missing results. | Text: possible publication bias discussed in Limitations section |
| RESULTS – Certainty of evidence | Not applicable | Present assessments of certainty (e.g., GRADE). | Discussion: not applicable (narrative review) |
| DISCUSSION – General interpretation | 16-17 | Interpret results in context of other evidence. | Discussion: interpretation of findings regarding efficacy and safety of techniques |
| DISCUSSION – Limitations of evidence | 18 | Discuss limitations of included evidence. | Discussion – notes observational designs, heterogeneity. |
| DISCUSSION – Limitations of review processes | 18 | Discuss limitations of review methods. | Discussion: clinical implications and recommendations for future research |
| DISCUSSION – Implications | 17-18 | Discuss implications for practice, policy, and research. | Discussion – calls for standardization, future research. |
| OTHER INFORMATION – Registration | Not applicable | Provide registration information for the review. | Not registered |
| OTHER INFORMATION – Protocol | Not applicable | Indicate where protocol can be accessed. | Not available |
| OTHER INFORMATION – Support | 21 | Describe sources of funding and role of funders. | Acknowledgements: no funding or institutional support stated. |
| OTHER INFORMATION – Competing interests | 21 | Declare competing interests. | Conflicts of interest: none declared |
| OTHER INFORMATION – Data, code, and materials availability | Not applicable | Report where materials can be accessed. | Extracted data presented in supplementary tables; PRISMA flowchart and NOS provided. |
