# Supplementary material for: Safety and Efficacy of Surgical Techniques in Treating Lipedema: Systematic Review
Source: Aesthet Surg J Open Forum. 2026 Feb 24;8:ojag039. doi: 10.1093/asjof/ojag039 (PMC13010320; doi:10.1093/asjof/ojag039)
Supplement: ojag039_Supplementary_Data [file ojag039_supplementary_data.zip › Supplemental Figure Legend.docx]

**Supplemental Figure Legend**

**Supplemental Figure 1**. Traffic-light plot of the risk of bias assessment of included studies according to the Newcastle–Ottawa Scale (NOS). Each domain (Selection: max 4 points; Comparability: max 2 points; Outcome/Exposure: max 3 points) is color-coded: green indicates the maximum score for the domain (low risk of bias), yellow indicates scores one point below the maximum (moderate risk of bias), and red indicates lower scores (high risk of bias). Total NOS scores and their respective risk categories (Low: 7-9 points; Moderate: 4-6 points; High: 0-3 points) are also shown.
