# Supplementary figures and images for: Safety and Efficacy of Surgical Techniques in Treating Lipedema: Systematic Review
Source: Aesthet Surg J Open Forum. 2026 Feb 24;8:ojag039. doi: 10.1093/asjof/ojag039 (PMC13010320; doi:10.1093/asjof/ojag039)

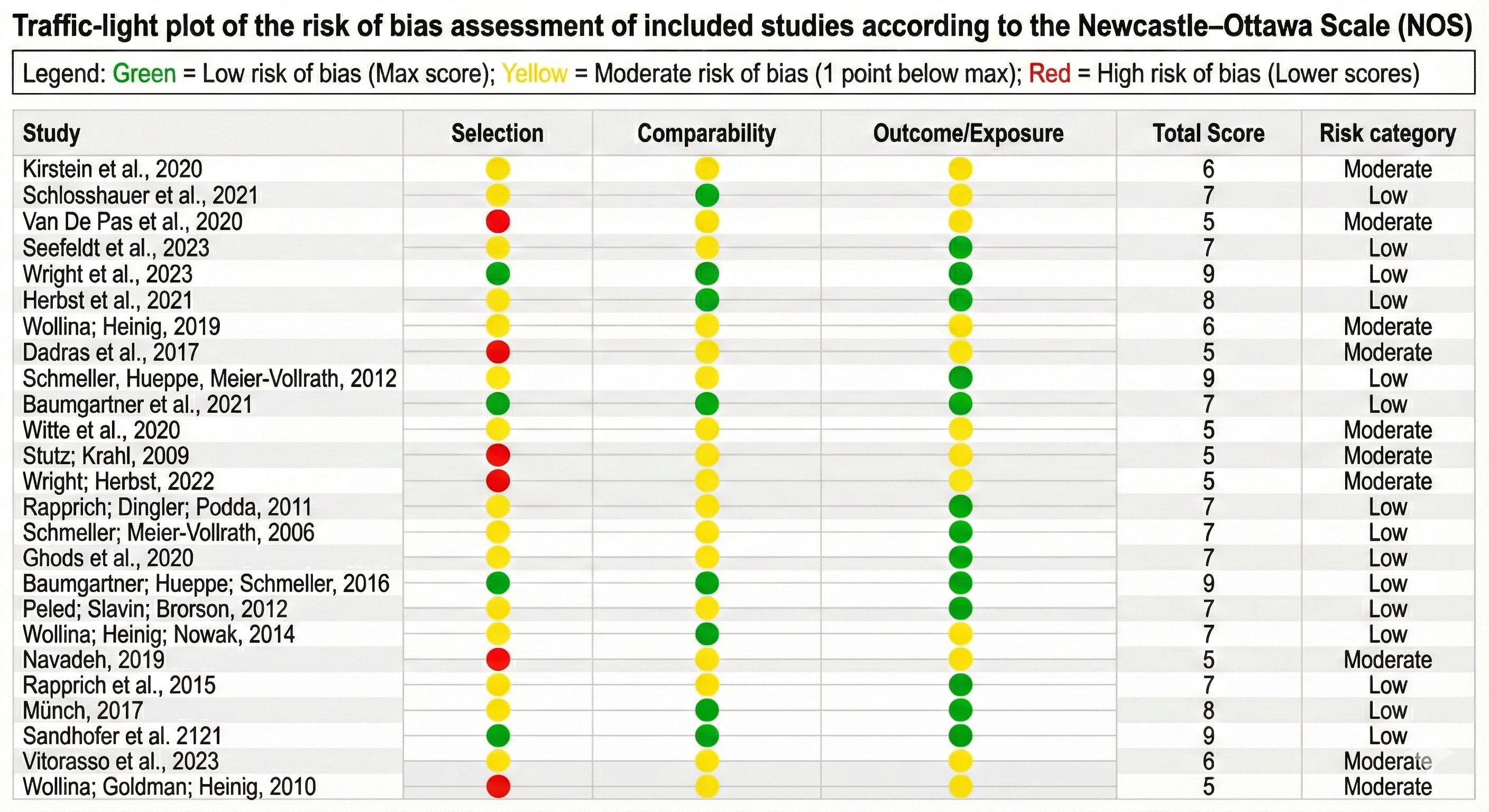

Supplement: ojag039_Supplementary_Data [file ojag039_supplementary_data.zip › Supplemental Figure 1.png]
